# Emergency Fixation of an Open Distal Phalanx Fracture Using an 18-Gauge Hypodermic Needle: A Case Report

**DOI:** 10.7759/cureus.115303

**Published:** 2026-08-27

**Authors:** Aghamurad Barkhudarli, Nurullah Aydogdu, Alican Baris, Muhammed Yusuf Afacan

**Affiliations:** 1 Department of Orthopaedics and Traumatology, Istanbul Physical Therapy and Rehabilitation Training and Research Hospital, Istanbul, TUR

**Keywords:** distal phalanx fracture, emergency department, hypodermic needle, nail-bed injury, open fracture, resource-limited care

## Abstract

Open distal phalanx fractures with nail-bed injury require prompt treatment of both the fracture and the nail-unit soft tissues. Kirschner-wire fixation is commonly used when an unstable fracture requires stabilization, but operating-room access and fluoroscopy may be delayed or unavailable in high-volume or resource-constrained emergency settings. Published series suggest that hypodermic-needle fixation can be an alternative in selected distal phalanx fractures, although the evidence remains limited. A 49-year-old man with hearing impairment sustained a door-crush injury to the dominant left middle finger, producing an open distal phalanx fracture with nail-bed laceration. Because timely operating-room access was not available, definitive management of the open injury was performed in the emergency department, with temporary fracture stabilization using a hypodermic needle. Under local anesthesia and sterile conditions, the open injury was irrigated and debrided, nail trephination and nail-bed repair were performed, and the fracture was reduced and stabilized with a sterile 18-gauge (38-mm) hypodermic needle. The wound was closed primarily, a finger splint was applied, tetanus prophylaxis was administered, and oral amoxicillin-clavulanate was prescribed for seven days. Immediate radiographs confirmed acceptable alignment. The needle was removed at four weeks, followed by gentle range-of-motion exercises. At six weeks, alignment and soft-tissue healing were maintained, fingertip sensibility remained intact, distal interphalangeal motion had returned, and no infection, fixation failure, or secondary operation was required. This case demonstrates the feasibility of hypodermic-needle fixation as a resource-contingent option in a carefully selected open distal phalanx fracture when conventional operative resources are not immediately available. This approach should not be interpreted as a substitute for standard operative fixation when formal surgical management is indicated.

## Introduction

The close anatomical relationship between the distal phalanx and nail bed means that fracture and nail-unit injury frequently coexist. Distal phalanx fractures are common consequences of crush trauma and are frequently accompanied by injuries of the nail plate, nail bed, and fingertip soft tissues. Management is therefore directed not only toward bony alignment but also toward preservation of the nail unit, sensibility, soft-tissue coverage, and distal interphalangeal (DIP) joint function [1,2]. Open injuries require prompt wound assessment, irrigation and debridement, appropriate nail-bed management, and fracture stabilization when displacement or instability cannot be controlled by splinting alone [2,3].

Kirschner wires remain a familiar fixation method for unstable distal phalanx fractures; however, fluoroscopy, a power driver, and operating-room availability are not universally or immediately accessible. This limitation is relevant in crowded emergency departments and becomes more important during disasters, mass-casualty situations, or other resource-constrained conditions. Prior studies have reported fixation with disposable hypodermic needles, including comparative cohorts and a small case series, with generally favorable union and functional outcomes in selected patients [4-6].

The concept is also transferable across small-bone injuries: our group previously described emergency department fixation of an open hallux fracture-dislocation with hypodermic needles when specialized equipment and fluoroscopy were unavailable [7]. The present report does not claim that hypodermic-needle fixation is new; rather, it contributes a transparently documented case report prepared in accordance with the CARE (CAse REport) guideline, which is an example of single 18-gauge needle stabilization integrated with definitive open-fracture and nail-bed treatment in the emergency department. A focused review of the most directly relevant literature is included to define where this approach may, and may not, fit in contemporary practice. Accordingly, the objective of this report is to describe the technical feasibility and short-term clinical outcome of single 18-gauge hypodermic-needle stabilization as one component of comprehensive emergency department management of an open distal phalanx fracture with nail-bed injury when conventional operative resources are not immediately available, and to place this approach in the context of the directly relevant literature.

## Case presentation

**Figure 1:**
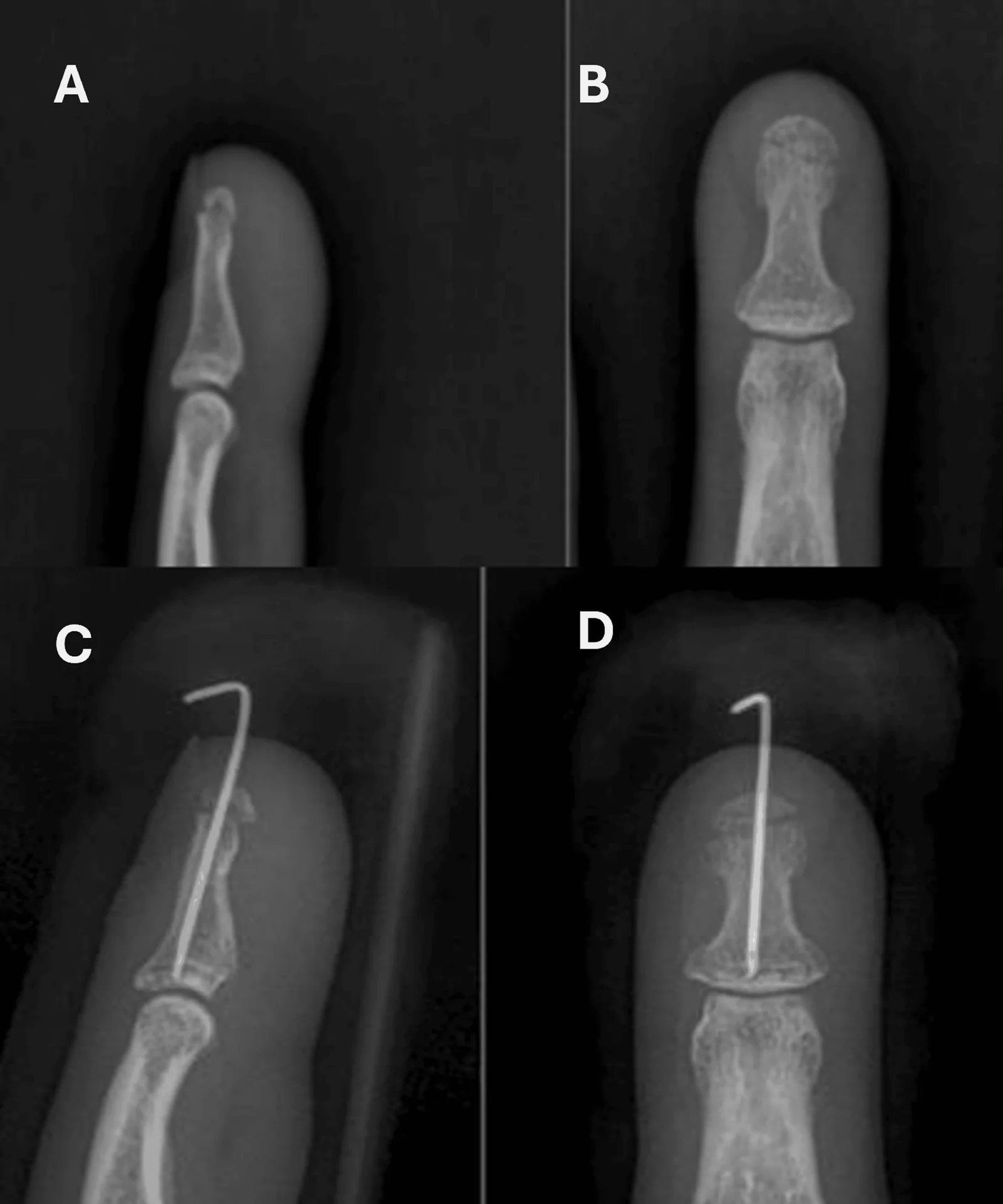
Plain radiographs of the dominant left middle finger. (A) Pre-intervention lateral view. (B) Pre-intervention anteroposterior (AP) view showing the open distal phalanx fracture. (C) Immediate post-procedure lateral view. (D) Immediate post-procedure AP view demonstrating anatomical reduction and stabilization achieved with a single 18-gauge hypodermic needle.

A 49-year-old man with hearing impairment presented to the emergency department after a door-crush injury to the dominant left middle finger. Examination demonstrated pain, deformity, an open distal phalanx fracture communicating with a nail bed laceration, and injury of the nail unit. Distal perfusion and sensibility were intact, and no neurovascular deficit was identified. No tendon deficit or extensive soft-tissue loss was documented. Initial anteroposterior (Figure 1B) and lateral radiographs (Figure 1A) confirmed the distal phalanx fracture.

The injury required prompt open-fracture and nail-unit care in addition to stabilization. Because timely operating-room access was not available in the setting of emergency department workload, management was undertaken in the emergency department by the treating orthopedic team. The patient was informed that a sterile hypodermic needle would be used off-label as a temporary fixation device, that this was a resource-contingent alternative to conventional K-wire fixation, and that risks included infection, loosening or breakage, loss of reduction, and the possibility of later operative treatment. Alternative management, including delayed formal operative fixation, was discussed, and written informed consent was obtained. Table 1 shows the timeline of clinical and radiographic events and management.

**Table 1:** Timeline of clinical and radiographic events and management. DIP: distal interphalangeal joint; ED: emergency department; ROM: range of motion

Time point	Clinical/radiographic findings	Management and outcome
Presentation	Open distal phalanx fracture of dominant left middle finger with nail-bed laceration; neurovascular examination intact.	ED decision after consent; local anesthesia; sterile open-fracture and nail-unit management.
Day 0	Immediate radiographs showed acceptable alignment after fixation.	Irrigation/debridement, trephination, nail-bed repair, reduction, single 18G needle fixation, primary closure, splint, tetanus prophylaxis, seven-day oral amoxicillin-clavulanate.
Approximately two weeks	Alignment maintained; soft-tissue healing progressing.	Continued protection and close outpatient surveillance.
Approximately three weeks	Clinical photographs obtained with needle still in situ; healing nail/soft tissues documented.	No acute post-procedure clinical photograph had been obtained.
Four weeks	Clinical/radiographic stability maintained.	Needle removed; gentle ROM begun after adequate soft-tissue healing.
Six weeks	Maintained alignment, healed soft tissues, maintained fingertip sensibility and DIP motion.	No infection, fixation failure, or secondary surgery during reported follow-up.

Under local anesthesia and sterile field preparation, the open wound was thoroughly irrigated and debrided. A subungual hematoma was decompressed by nail trephination. The nail plate was then carefully elevated to expose the nail-bed laceration. The nail bed was repaired with 3-0 absorbable suture, after which the nail plate was repositioned beneath the eponychial fold. The open fracture was subsequently reduced and stabilized with a sterile 18-gauge hypodermic needle. A sterile pink-tipped 18-gauge, 38-mm hypodermic needle (1.20 × 38 mm; Ayset Tıbbi Ürünler San. A.Ş., Adana, Türkiye) was then advanced as a temporary internal fixation device to maintain alignment. After confirmation of satisfactory stability, the soft-tissue wound was closed primarily, and a protective finger splint was applied. Tetanus prophylaxis was administered. Oral amoxicillin-clavulanate was prescribed for seven days as part of the treating team’s open-injury protocol. Distal neurovascular status remained intact after the procedure.

**Figure 2:**
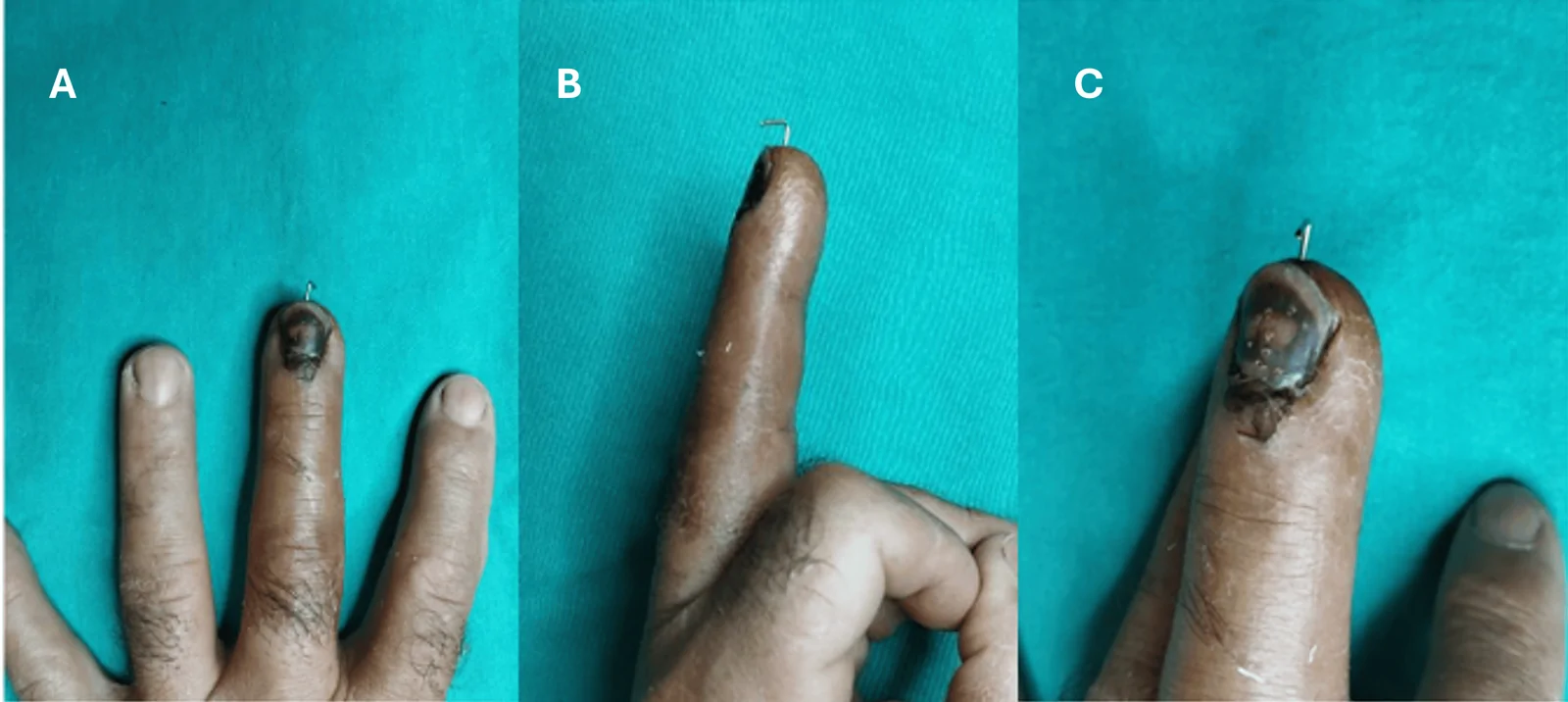
Clinical appearance approximately three weeks after emergency department treatment. (A) Dorsal view of the hand showing the affected middle finger with the fixation needle in situ; (B) lateral profile view demonstrating finger alignment and needle position; and (C) close-up dorsal view documenting interval healing of the nail unit and surrounding soft tissues. (Note: These photographs reflect follow-up healing and are not immediate post-procedure images).

Immediate post-procedure anteroposterior (Figure 1D) and lateral radiographs (Figure 1C) demonstrated acceptable reduction and needle position. An acute post-procedure clinical photograph was not obtained. The clinical photographs available for this report were taken at approximately three weeks after the intervention and therefore are presented and labeled as three-week follow-up images rather than immediate postoperative images (Figures 2A-2C).

The patient was reviewed at approximately 10-day intervals. Serial examinations showed progressive soft-tissue healing, an intact nail-bed repair, and no clinical evidence of pin-tract infection, deep infection, secondary displacement, or fixation failure. Follow-up radiographs were obtained after the procedure and at approximately two weeks, four weeks, and six weeks. The fixation needle was removed at four weeks after clinical and radiographic stability had been confirmed. Gentle range-of-motion exercises were initiated after removal and adequate soft-tissue healing.

**Figure 3:**
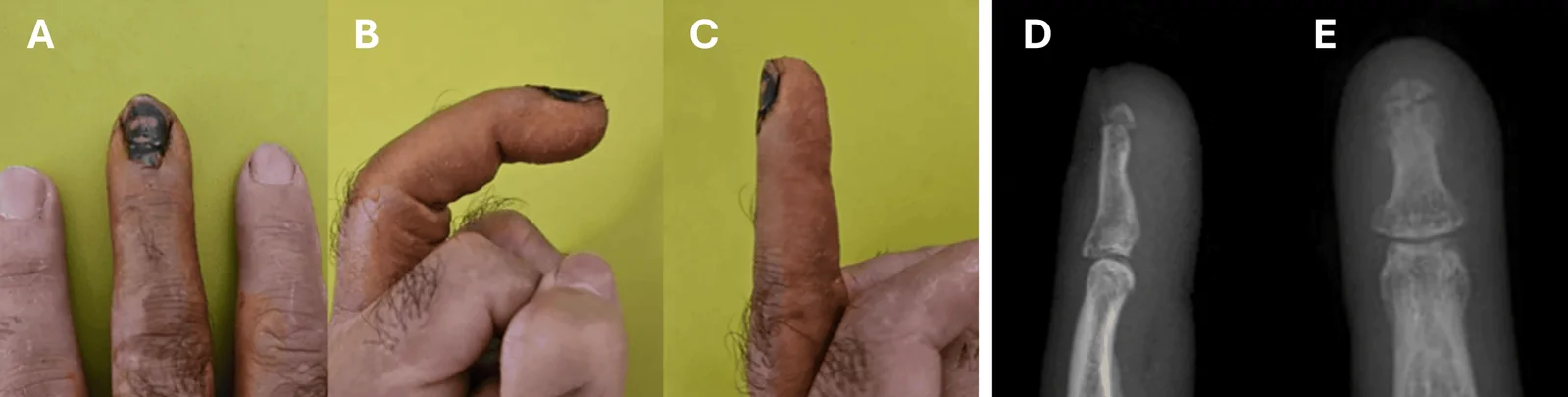
Comprehensive six-week follow-up and multi-planar assessment. Clinical photographs (left panel) and corresponding radiographs (right panel) at six weeks post-injury and two weeks following needle removal. Healed soft tissues and nail plate are evident in all clinical views. Active joint motion is demonstrated. Radiographic alignment is anatomical in both planes. (A) Dorsal view of fisted hand. (B) Proximal lateral view of fisted hand. (C) Close-up lateral profile view of the third digit fingertip. (D) Standard lateral radiograph. (E) Standard anteroposterior (AP) radiograph.

At the six-week review, the soft tissues had healed (Figures 3A-3C), fracture alignment remained acceptable (Figures 3D-3E), fingertip sensibility remained intact, and active DIP joint motion was present. No secondary surgical intervention was required during the reported follow-up. A formal patient-perspective narrative was not prospectively collected (Table 1).

## Discussion

The central clinical issue in this case was not fixation in isolation. The patient had an open distal phalanx fracture associated with nail-bed injury, so successful emergency treatment required coordinated management of contamination, the open wound, the nail unit, fracture alignment, immobilization, and follow-up. Nail trephination and nail-bed repair were therefore performed together with irrigation/debridement and stabilization. This distinction is important because a percutaneous fixation device cannot compensate for inadequate open-fracture or soft-tissue care [1-3].

Previous studies support the feasibility of hypodermic-needle fixation but differ substantially in technique and treatment setting. Senesi et al. used two crossed 23-gauge needles under fluoroscopic control, whereas Van Royen et al. performed hypodermic-needle fixation in an emergency treatment room without fluoroscopy and reported similar healing outcomes to K-wire fixation, although needle loosening was more frequent [4,5]. Takamoto et al. subsequently described a needle-in-needle technique in eight open distal phalanx fractures [6]. In contrast, the present case used a single 18-gauge needle as temporary stabilization within comprehensive emergency department management of an open distal phalanx fracture with associated nail-bed injury. Our previously reported hallux fracture-dislocation further illustrates the potential cross-anatomic applicability of readily available hypodermic needles for selected small-bone injuries in resource-constrained settings (Table 2) [7].

**Table 2:** Summary of directly relevant studies of hypodermic needle fixation and context of the current case. This focused review summarizes directly relevant studies [4-6] in which disposable hypodermic needles were utilized for distal phalanx fixation, with an emphasis on key outcomes and the clinical environment (ED or OR). A previously published case of hallux proximal phalanx fracture [7] was also included to demonstrate the cross-anatomic feasibility of this technique. The current case contributes a unique, transparent, and multi-faceted chronology demonstrating success in a single-18G construct for a combined open injury, framed within a complete, CARE (CAse REport)-compliant ED treatment bundle. ED: emergency department; G: gauge; ROM: range of motion; DIP: distal interphalangeal; OR: operating room; K-wires: Kirschner wires

Study	Design/setting	Fixation approach	Key findings	Relevance to present report
Senesi et al., 2020 [4]	Retrospective comparative cohort; 60 distal phalanx fractures; needle group in ED surgical room.	Two crossed 23G hypodermic needles vs crossed K-wires; fluoroscopy used in both groups.	Needle group had satisfactory union/ROM outcomes; no infections; selected tuft/shaft fractures favored.	Supports feasibility of small-gauge needle fixation, but differs from the present single 18G construct.
Van Royen et al., 2021 [5]	Retrospective comparative cohort; 49 distal phalanx fractures.	Hypodermic needle in emergency treatment room without fluoroscopy vs K-wire in OR with fluoroscopy.	Similar healing/union; no infections; lower cost; more loosening with needles.	Most directly supports a resource-contingent ED pathway without fluoroscopy.
Takamoto et al., 2022 [6]	Case series; eight open distal phalanx fractures.	Needle-in-needle disposable hypodermic-needle pinning.	Successful stabilization and functional recovery reported in a small series.	Demonstrates application specifically in open distal phalanx injuries when standard instruments are unavailable.
Afacan et al., 2026 [7]	Case report; open hallux proximal phalanx fracture-dislocation in the ED.	21G and 18G hypodermic needles without fluoroscopy.	Union and pain-free full ROM; resource-limited/disaster rationale emphasized.	Provides cross-anatomic proof of concept for emergency small-bone stabilization with readily available needles.
Present case	CARE-compliant single case; open distal phalanx + nail-bed injury.	Single 18G (38 mm) hypodermic needle plus definitive open-fracture/nail-bed treatment in the ED.	Maintained alignment, healed soft tissues, restored sensibility and DIP motion at six weeks; no secondary surgery.	Adds transparent chronology and emphasizes that needle fixation is one component of a complete ED treatment bundle.

The contribution of the present report is not the novelty of hypodermic-needle fixation itself, but the integration of a single 18-gauge needle into a complete emergency department treatment pathway that included open-fracture care, nail-bed management, splint protection, and close follow-up. This resource-contingent principle is consistent with our previously reported use of hypodermic needles for a hallux fracture-dislocation [7].

This approach should not be extrapolated to all open distal phalanx fractures. A hypodermic needle is a hollow, non-orthopedic implant used off-label and should not be assumed to have mechanical behavior equivalent to a solid K-wire. Based on the limitations of the available literature and our clinical experience, we propose that this technique be reserved for carefully selected injuries in which satisfactory reduction and stability can be achieved with a simple construct and supplemental splinting. Features such as substantial contamination, major soft-tissue or bone loss, associated tendon or neurovascular injury, complex intra-articular involvement, marked comminution, or inability to obtain and maintain reduction should prompt consideration of formal operative management rather than reliance on this technique. These factors should be regarded as practical selection considerations proposed by the authors rather than validated contraindications specific to hypodermic-needle fixation. Needle loosening remains a documented concern in the comparative literature [5].

Antibiotic practice in open distal phalanx fractures remains debated. In this patient, a seven-day course of oral amoxicillin-clavulanate was chosen by the treating team. A systematic review and meta-analysis did not demonstrate a statistically significant reduction in superficial infection with prophylactic antibiotics in open distal phalanx fractures and emphasized prompt irrigation and debridement [8]. Accordingly, the favorable outcome in this case should not be attributed to antibiotics alone; meticulous open-fracture and nail-bed management, stable alignment, splint protection, and surveillance were likely more fundamental components of care. Antibiotic decisions should follow local open-fracture protocols and patient-specific contamination and risk factors.

More recent comparative data suggest that fixation may be beneficial in selected unstable or displaced open distal phalanx tuft fractures [9]. However, because that study specifically evaluated tuft fractures, its findings should not be directly generalized to other distal phalanx fracture morphologies or interpreted as evidence of superiority of hypodermic-needle fixation over standard K-wire fixation.

Contemporary reviews further emphasize that distal phalanx and fingertip injuries require treatment tailored to fracture stability and the associated nail and soft-tissue injury. De Ruiter et al. highlighted that unstable or substantially translated fractures may warrant operative stabilization and that nail-bed management should be individualized according to the extent of nail-unit injury [10]. More specifically, a recent systematic review of 24 studies involving 915 open distal phalanx fractures emphasized timely debridement as a central component of treatment and highlighted the continuing lack of standardized management protocols [11]. These data place the present case in a broader context and reinforce that its favorable short-term outcome should be interpreted as the result of comprehensive open-fracture and nail-unit management together with stable fracture fixation, rather than as an effect of the hypodermic needle alone.

This report describes a single patient with short-term follow-up and therefore cannot establish comparative safety, union rates, infection risk, or mechanical equivalence to K-wire fixation. The exact fracture morphology was not prospectively classified with a formal instability score, and no standardized patient-reported outcome measure or objective dynamometric sensory test was recorded. An immediate post-procedure clinical photograph was also unavailable; Figure 2 documents the three-week clinical appearance and has been labeled accordingly to avoid a misleading chronology. These limitations reinforce the need for prospective case series and comparative studies with predefined eligibility criteria, standardized radiographic and patient-reported outcomes, explicit adverse-event surveillance, and longer follow-up. Future studies should directly compare hypodermic-needle and conventional K-wire fixation with respect to maintenance of reduction, union, infection, implant-related complications, nail outcomes, functional recovery, and cost.

## Conclusions

In a carefully selected open distal phalanx fracture with nail-bed injury, emergency department irrigation/debridement, nail trephination and repair, reduction, and temporary stabilization with a single 18-gauge hypodermic needle resulted in maintained alignment and satisfactory short-term clinical recovery without secondary surgery. This case demonstrates the feasibility of single-needle stabilization as a resource-contingent option in carefully selected circumstances when operating room access, fluoroscopy, or conventional pinning equipment is not immediately available. It should not be considered a substitute for formal operative management when injury complexity or instability warrants standard surgical treatment.
